# The impact of total factor mobility on rural-urban symbiosis: Evidence from 27 Chinese provinces

**DOI:** 10.1371/journal.pone.0294788

**Published:** 2023-12-14

**Authors:** Xiangmei Zhu, Shaohua Guo, Hui Yuan

**Affiliations:** School of Economics and Management, North University of China, Taiyuan, Shanxi, China; Khwaja Fareed University of Engineering & Information Technology, PAKISTAN

## Abstract

The rational flow and optimal allocation of urban and rural factors is the key to solving the problem of unbalanced and insufficient urban and rural development. This study draws on the theory of ecological symbiosis to examine the mechanism of factor flow and urban-rural symbiosis. It analyzes panel data from 27 Chinese provinces and autonomous regions between 2010 and 2020 to empirically demonstrate the influence of labor, capital, technology, and land mobility on urban-rural symbiosis. The study found that: (1) The relationship between the total factor flow and urban-rural symbiosis is U-shaped nonlinear, and the time when cities feed the development of rural areas has come; (2) The impact of labor factor flow on urban-rural symbiosis exhibits an inverted U-shaped relationship, the impact of capital factor flow displays a positive U-shaped relationship, the impact of land factor flow shows an inverted U-shaped relationship, and the impact of technical factor flow on urban-rural symbiotic development is not significant. (3) The factor flow exerts a region-oriented impact on the level of urban-rural symbiosis. In developed areas, total factor flow does not affect urban-rural symbiosis, but the level of labor flow and the urban-rural symbiosis demonstrates an inverted U-shaped relationship, the flow of technological factors has a U-shaped impact on the urban-rural symbiosis level, the flow of land factors and the urban-rural symbiosis show an inverted U-shaped relationship, and the flow of capital factors has no impact on the urban-rural symbiosis relationship. In underdeveloped areas, the impact of the total factor flow on urban-rural symbiosis shows a significantly positive U-shaped characteristic, the labor mobility level and urban-rural symbiosis show an inverted U-shaped relationship, the flow of capital factors has a U-shaped impact on the urban-rural symbiosis level, and the flow of lands and technology does not have a remarkable impact on urban-rural symbiosis in underdeveloped areas.

## 1. Introduction

City and township, as two major spatial forms of human production and life, are a community with a shared future in which both of them accompany, strengthen each other and integrate. China always attaches importance to the coordinated development of urban-rural relationships. Since the start of the 21st century, the urban-rural development strategy of China has been adjusted and reformed many times, ranging from " urban-rural coordination" through " urban-rural unity" to "urban-rural integration" which results in the improvement of urban-rural relationships. However, the dual order of urban-rural division has not been fundamentally resolved, and the unbalanced development of urban and rural areas and the inadequate development of rural areas remain a prominent contradiction in China’s economic and social development. Among them, the imbalance in the flow of urban and rural factors is the primary obstacle to the coordinated development of urban and rural areas in China. For a long time, labor, capital, technology and other factors of production have found their way from rural areas to cities. That explains why urban areas enjoy more resources, Agricultural development stagnates, rural areas lack vitality and the population in the countryside ages [1]. The 20th National Congress of the Communist Party of China emphasized "adhering to the integrated development of urban and rural areas and ensuring the unfettered flow of urban and rural factors"; The "Opinions on Establishing and Improving the Institutional Mechanism and Policy System for Urban-Rural Integrated Development" and the "14th Five-Year Plan for Promoting Agricultural and Rural Modernization" issued by the CPC Central Committee and the State Council also clearly pointed out that it is necessary to promote the unimpeded flow of urban and rural capital, lands, talents, information and other aspects, pursue the circulation of urban and rural factors at the macro level, and advance the integrated development of urban and rural areas. These top-level plans demonstrate factor flow is highly relevant for the benign development of urban-rural relations. The urban-rural relationship which involves politics, economy, culture, society, ecology and many other fields, is a complex system engineering. In this case, traditional economics, geography, sociology and other single perspectives tell only part of the story of the relationship. To provide a new explanatory framework for realizing the virtuous, lasting circle of the urban and rural symbiosis of co-prosperity and harmony, we can take urban and rural areas as two important organic groups of the human ecosystem, view the complex interaction fields as subsystems, review and identify the symbiotic development mechanism of urban and rural areas. Based on this, this paper draws on the theory of ecological symbiosis, constructs an urban-rural symbiosis model, measures the level of urban-rural symbiosis in China, and conducts empirical research on the urban-rural symbiosis mechanism from the perspective of the total factor flow, to offer theoretical guidance for identifying the practical path of urban-rural reciprocal symbiosis.

## 2. Literature review

### 2.1. Symbiosis theory

"Symbiosis" was first proposed by German biologist Anton de Berry, who pointed out that "symbiosis is a permanent material connection between different organisms", consisting of three elements: symbiotic unit, symbiosis mode and symbiotic environment [2]. The symbiotic unit is the basic energy production and exchange unit that constitutes a symbiote or symbiotic relationship, and it is the basic material condition for the formation of a symbiote. The symbiotic mode refers to the form of the combination of symbiotic units, which can be divided into parasitic, commensal symbiosis and reciprocal symbiosis in terms of the behavior. The symbiotic environment is the external condition for the existence and development of the symbiotic model, and all factors outside the symbiotic unit constitute the symbiotic environment [3, 4]. Generally speaking, symbiosis refers to the interdependent and co-evolved relationship between symbiotic units in a certain symbiotic environment through the dynamic exchange of matter, energy and information, according to a certain symbiotic mode. It can be used to analyze the interaction between different subjects in the system and the dynamic relationship between the subject and the environment [5], scholars’ explorations of symbiosis theory in biology have involved symbiotic relationships between different species. These include insect-microbe interactions , symbiont dynamics and phylogeny, the effect of symbionts on the hostand the role of symbionts in species evolution and community structure. In addition to this, it can provide a new thinking and interpretation framework for the survival, prosperity and development of human society and living space at a higher level, and has been widely used in the fields of political science, ethnology, economics, and sociology. The complexity, pluralism and dynamics of the urban-rural giant system significantly align with the symbiosis theory. Therefore, this paper uses the symbiosis theory to interpret the urban-rural relationship and explain the interaction and evolution law between urban and rural areas at a higher level.

### 2.2. Urban-rural symbiosis

Western developed countries have basically achieved urban-rural integration and rural modernization [5], and the concept of symbiosis is implied in many classic discussions. For example, the "utopian" society conceived by More, the idea of "equality between urban and rural society" expounded by Saint-Simon, the "harmonious society" imagined by Fourier, the future social development concept of "urban-rural integration" jointly proposed by Marx and Engels, the "pastoral city" theory of integrated urban and rural development proposed by Howard and the unified development concept of urban driving rural areas proposed by Mumford all reflect the connotation of the ideal of reciprocal symbiosis between urban and rural areas to varying degrees [4, 6, 7]. Many western scholars have put forward the path of urban-rural symbiosis in developing countries from the perspectives of labor transfer [8], government responsibility [9], cities driving villages [10], and industrial promotion [11, 12], which has important enlightening significance for the speculation of urban-rural relations in China. Based on the reality of China’s typical urban-rural dual pattern, Chinese scholars have carried out rich research on the theoretical connotation, relationship evolution, influence mechanism and improvement path of urban-rural symbiosis in China. Among them, scholars have the same views on the theoretical connotation of urban-rural symbiosis, believing that the core of urban-rural symbiosis aims to take equality and justice as the development concept and mutually beneficial cooperation as the development path, promote the common prosperity of urban and rural areas, and achieve symbiotic, reciprocal and reasonable coexistence between urban and rural areas [13], emphasizing the dynamic evolution of urban-rural relations from skewness to normal [4]. China’s urban-rural governance follows this gradual process, from "parasitic" governance, to "commensal symbiosis" governance, "asymmetric reciprocal symbiosis" governance, and "symmetrical reciprocal symbiosis" "Governance model [13], China’s urban-rural relations have experienced a stage of differentiation, confrontation, integration and unity [14]. However, due to the long-term existence of barriers such as the land system and the household registration system, the imbalance in urban and rural factors, public services and resource allocation is still serious [1]. Many scholars focus on the influencing factors of urban-rural symbiosis, looking for breakthroughs in the further benign development of urban-rural relations, pointing out that the free flow of urban-rural factors is the material guarantee for rural development and an effective way to break the imbalance pattern of urban-rural development [6, 15], in addition, the level of marketization [16], transportation infrastructure [17–19], spatial structure [7, 20] and so on also have an impact on urban and rural development. When it comes to the practical path of urban-rural symbiosis, some scholars take urban and rural areas as the basic symbiotic units, and respectively discuss the internal logic of urban and rural spatial optimization and governance [20], education integration [21–23], public service development mechanism [24], etc., and put forward specific strategies for the advancement of urban and rural cultural integration [25], ecological construction [26], financial relationship evolution [27], tourism resource interaction [28], etc., as a way to achieve mutually beneficial symbiosis, harmonious progress and sustainable development of urban and rural symbiosis [29].

### 2.3. Factor flow and urban-rural relations

Factors are the necessary basic units that power the existence of objective things and maintain their movement [30], and the factors in the study of urban-rural relations specifically refer to production factors such as labor, lands, capital, and technology, and factor flow refers to the spatial migration of these production factors between urban and rural areas or the transformation of ownership to maximize efficiency and utility [31]. The flow of land factors refers to the result of the competitive allocation of land between two uses (agricultural use and construction utilization) and two types of ownership (collective ownership and state ownership), which is mainly reflected in the transformation of planning use and the change of land ownership. The main channels for the flow of land elements are market entry, increase and decrease linkage and land acquisition [32]. Labor factor flow refers to the process of labor force transfer between urban and rural areas with the main purpose of changing the type of employment and improving income. Capital factor flow mainly refers to the flow between urban and rural areas through fiscal, price, financial and other channels due to government transfer payments, differences in financial market returns and the need for risk management [33]. The flow of technological elements involves technology transfer and knowledge diffusion. Western scholars researched factor flow and urban-rural relations and put forward many mature theories and views at an early date. Neoclassical economics believes that if there is no obstacle to the flow of factors in the market, the convergence of labor productivity between agriculture and non-agricultural industries can be achieved through the transfer of agricultural surplus labor [34, 35]; The new dual economic growth theory points out that the lack of sufficient free flow of production factors between agriculture and non-agricultural sectors will adversely affect the dissolution of the urban-rural dual structure [36]. In addition, Kuznets (1955) proposed the inverted U-curve of economic growth and income gap, and applied it to the study of the relationship between urban-rural differences and economic growth rates, pointing out that the inverted U-curve from segregation to the free flow of urban and rural factors is the reason for the emergence of the inverted U curve [37]. Later, Harris and Todaro (1970) proposed a model of urban-rural labor migration, exploring the correlation between labor factor flow and the urban-rural gap [38, 39]. The essential pursuit of urban-rural symbiosis is to realize the sharing of social development achievements between urban and rural areas [40], and these studies reveal that the improvement of urban-rural symbiosis focuses on removing the institutional and mechanical barriers of factor flow between urban and rural areas, and strengthening the free flow and equal exchange of factors between urban and rural areas. In recent years, Chinese scholars have conducted rich research on the relationship between factor flow and urban-rural development, mainly focusing on two aspects: one is to focus on the type, direction, degree of freedom and intensity of urban-rural factor flow [14], and to discuss the impact on urban-rural relations. Urban and rural factors have developed from urban to rural or from rural to urban one-way flow to two-way and circular flow of various factors between urban and rural areas [15]. The one-way flow of rural production factors to cities is an important cause of rural economic decline. Therefore, promoting the two-way flow of urban and rural factors is an important way to achieve urban-rural symbiosis [41]. According to the different roles of the government and market players, production factors have experienced free, directive and restricted flow types, and the higher the intensity of factor flow, the closer the connection between urban and rural areas [14]; Second, from the perspectives of economics and geography, measure the impact of specific single elements on urban and rural development. The influence of the flow and agglomeration of single factors such as human capital [42], finance [27], and land [32] on urban-rural relations was explored, and the nonlinear relationship between single-factor flow, urban-rural income gap and urban-rural economic integration development was verified [43], but few studies discussed the nonlinear characteristics of factor flow and urban-rural reciprocal coexistence from the perspective of all factors.

The above literature has certain explanatory power in the research of factor flow and urban-rural symbiotic relationship, but there are several shortcomings. First of all, the measurement of the development level of urban-rural relations is mostly concentrated on the level of urban-rural coordination, urban-rural coordination, and urban-rural linkage, which is difficult to reflect the overall picture of the complex system of urban and rural areas, and urban and rural should be an ecological symbiotic system, and there is a dynamic and mutually promoting relationship of interaction, but there is little literature to quantitatively evaluate the level of urban-rural symbiosis. Secondly, the research on factor flow and the urban-rural relationship is currently based on the perspective of a single factor, and scholars emphasize that the core of the urban-rural symbiotic relationship is the free flow of labor, capital, technology and other factors, but lack a comprehensive perspective of all-factor flow, and it is difficult to accurately grasp and comprehensively examine the formation mechanism of the evolution of the urban-rural symbiotic relationship. Third, the nonlinear impact of factor flow on urban and rural development is limited to the economic dimension dominated by the income gap, and there is little literature to deeply explore the nonlinear impact of factor flow on the all-round symbiotic development of urban and rural areas in economic, social, political, cultural and ecological aspects. Therefore, based on the theory of ecological symbiosis, this paper constructs a comprehensive evaluation index system from the aspects of "political sharing, economic co-prosperity, social co-construction, cultural integration, and ecological co-governance" to measure the level of urban-rural symbiosis in China, and empirically tests the evolution mechanism of urban-rural symbiosis in China from the perspective of the flow of all factors such as labor, capital, technology, and land, to enrich the achievements in the field of urban-rural relations and provide practical guidance for building fully balanced urban-rural relations and realizing urban-rural co-prosperity and co-prosperity and sharing.

## 3. Theoretical hypothesis

China’s urban and rural factors have significant dual characteristics, rural areas have land, labor and other production factors, and cities and towns are the main body of capital, technology and other factors [35]. For a long time, under the influence of the traditional dual economic system and urban bias policy, to give play to the advantages of agglomeration economy, production factors such as labor, capital, technology, and land have shown the characteristics of one-way flow from rural to urban [35, 43]. Excessive loss of rural factors will inhibit rural revitalization [44], which in turn affects urban-rural economic integration [45], resulting in a widening income gap between urban and rural areas [17]. Under the influence of government policies, market monopoly, systems and other factors, the flow of urban and rural production factors in China has formed a mismatch between agriculture and non-agriculture, including labor misallocation and capital misallocation [35]. This all-factor urban bias leads to rapid urban development, while the countryside is gradually hollowed out [46], and the gap between urban and rural development is gradually widening. Urban and rural complementarity, industry and agriculture promote each other, and it is difficult to play the coupling and coordination role of the government and the market [39], which is not conducive to the improvement of the level of urban-rural symbiosis. However, the production factors continue to flow to the city, which will cause excessive urban agglomeration of factors, the decline in urban production efficiency, and the congestion effect [43], so when the production factors agglomerate in cities and towns increase to a certain critical value, the "traction effect" of factor marketization begins to occupy a dominant position [16, 44], while the level of total factors increases, the marketization process of technology, capital and other factors accelerates [16], and the factor agglomeration effect caused by the increase of agricultural capital input and the improvement of agricultural modernization technology begins to play [35]. Rural areas gradually realize the improvement of total factor productivity from technological innovation to institutional innovation [16]. In addition, starting from the economic subject, the essence of income is the remuneration of production factors, and the urban-rural allocation of production factors is the decisive factor in the evolution of urban and rural income [43], a large number of rural people migrate to cities and towns, helping rural residents to obtain a wider range of employment opportunities, thereby promoting farmers’ income, and narrowing the urban-rural income gap [47], and population migration further increases the demand for corresponding factor flow allocation, which is conducive to agricultural modernization and new urbanization. Factor marketization can drive rural revitalization by promoting new urbanization [16], and the pace of coordinated urban-rural development accelerates, when the increase in the flow level of production factors narrows the development gap between urban and rural areas, thereby promoting urban-rural symbiosis. Based on this, this paper proposes hypotheses:

**Hypothesis 1(H1):** The influence of total factor flow on urban-rural symbiosis has U-shaped nonlinear

characteristics.

In addition, due to the different stages of industrialization, urbanization and economic development of various provinces and regions, the flow level of factors also shows the characteristics of regional differences [43]. The eastern region with location advantages has high factor flow efficiency, fast urban development speed, and economically developed cities which can feed the development of rural areas. While central and western regions are greatly affected by macro policies and institutional regulation, insufficient market-oriented flow capacity of factors, factor resources play a more agglomeration effect in cities, and rural development is limited, resulting in urban-rural integration showing the characteristics of unbalanced development between regions [45]. Based on this, this paper proposes hypotheses:

**Hypothesis 2(H2):** The impact of the total factor flow on the level of urban-rural symbiosis varies by region.

## 4. Study design

### 4.1. Model settings

To test the research hypothesis of this paper and empirically verify the influence of total factor flow on the level of urban-rural symbiosis, the following benchmark model is constructed:

$$CXGS_{it}=\beta_{0}+\beta_{1}QYS_{it}+\beta_{2}{\left(QYS_{it}\right)}^{2}+\beta_{3}C_{it}+\varphi_{\text{i}}+\mu_{\text{t}}+\varepsilon_{\text{it}}$$ (1)

In model (1), *i* represents the regional cross-sectional unit in the sample, *t* represents the year, *β*_*0*_ is the constant term of the model, *β*_*1*_*∼β*_*3*_ is the coefficient to be estimated for the corresponding variable, *φ*_*i*_ and *μ*_*t*_ represent the regional individual effect and time effect, respectively, and *ε*_*it*_ is the random disturbance term. *CXGS* is the urban-rural symbiosis level in the region, *QYS* is the total factor flow level in the region, and *C* represents the set of control variables affecting the urban-rural symbiosis level in the region. The square term of total factor flow is introduced in the model (1) to examine the possible nonlinear relationship between total factor flow and urban-rural symbiosis.

### 4.2. Variable picking

#### 4.2.1. Explanatory variables

Total factor flow (*QYS*). In the calculation of the index, this paper abstracts the main position of total factor flow into two major spaces, cities and villages, that is, the application of production factors is not urban or rural, and the proportion of factors in urban or rural areas is used to represent the level of factor flow. Since the nonlinear relationship between the core variables is discussed, the key to empirical evidence is to judge the quadratic coefficient symbol and significance of the total factor flow level, so the empirical conclusion of using the proportion of factors in rural areas and the proportion of factors in urban areas as the total factor flow level is the same. Given the availability of data, this paper uses the proportion of factors in rural areas to reflect the level of factor flow between urban and rural areas. Taking the flow of total factors as the first-level indicator, and the flow of labor, technology, capital and land factors as the second-level indicator, the entropy weight method is used to determine the weight and score. Among them, the flow of labor factors (labor) draws on the practices of Zhang Juntao, You Bin (2021) [39], Liu Xiaoguang, Zhang Xun et al. (2015) [48] and measures the proportion of employees in the primary industry, reflecting the level of rural labor after the transfer of labor to the secondary and tertiary industries in the process of industrialization and urbanization. Land flow refers to the transformation of rural homesteads, farmland, etc. into urban construction land and industrial land, drawing on the practice of Zhang Ying and Lei Guoping (2019) [49], using the ratio of urban built-up area to total land area to measure the flow of land elements, the larger the value, indicating that fewer rural factors are remaining after the factor flows to cities, which is a negative indicator; Capital factor flow refers to the practice of Li Junjie, Liang Hui et al. (2022) [50], and is measured by the proportion of agriculture-related loans; The flow of technical elements is measured by the degree of agricultural mechanization, and the specific indicator is the total power of agricultural machinery per capita [43]. Table 1 describes the specific assignment methods.

**Table 1:** Composite indicators of total factor flows.

Primary index	Secondary indicators	Assignment method	Attribute	Weight
Total factor flow	Factor mobility of labor	Number of employees in the primary industry/total number of employees	+	0.1922
	The flow of technical elements	Agricultural machinery general power/agricultural practitioners	+	0.5144
	Capital element flows	Balance of agriculture-related loans/balance of various loans	+	0.2220
	Land element flows	Area of built-up area of the city/total land area	-	0.0714

#### 4.2.2. Explained variable

Urban-rural symbiosis level (*CXGS*). The symbiotic relationship between urban and rural areas is a dynamic organic system formed by a continuous flow of capital, technology, labor and other factors in a certain symbiotic environment. Based on the concept of "political sharing, economic co-prosperity, social co-construction, cultural co-construction, and ecological co-governance", this paper constructs a comprehensive evaluation index system for an urban-rural symbiotic relationship, and uses the entropy value method to calculate the weight. Among them, political sharing refers to the rational allocation of political resources in urban and rural areas, including policy support, laws and regulations, municipal public facilities construction, public financial investment, social welfare, spatial layout and many other aspects, which require the government to rationally allocate resources. Economic co-prosperity refers to abandoning the concept of "sacrificing rural areas and developing cities", aiming at the mutual promotion and reciprocal symbiosis between urban and rural economies, narrowing the economic differences between urban and rural areas, and realizing common prosperity of urban and rural economies. Social co-construction mainly focuses on people’s livelihood issues, improves people’s livelihood level, and focuses on solving the problems of difficulty in seeing a doctor and finding employment; In addition, the construction of socialism with Chinese characteristics should develop towards informatization and digitalization, and the Internet is the foundation of informationization construction. At the cultural level, this paper mainly considers the difference between urban and rural educational standards, and measures the level of urban-rural cultural coordination from the differences in educational resources, book resources and cultural consumption. In the face of increasingly severe environmental problems, it is necessary to pay attention to ecological protection during urban and rural development, and this paper measures the ecological coordination level of urban and rural areas from the per capita green space area, per capita daily water consumption and per capita carbon emissions. Table 2 describes the specific assignment methods.

**Table 2:** Comprehensive indicators of urban-rural symbiosis.

Primary index	Secondary indicators	Assignment method	Weight
Political sharing	Social security coordination degree	Expenditure of basic medical insurance fund for urban workers / Expenditures from basic medical insurance funds for non-working urban and rural residents	0.0484
	Road area coordination degree	Per capita urban road area / Per capita road area in rural areas	0.1469
	Degree of coordination of public expenditure	Urban general public financial expenditure / Rural general public financial expenditure	0.0183
	Urbanization rate	Urban population / rural population	0.0960
Economic co-prosperity	Income difference between urban and rural residents	Per capita disposable income of urban residents / Per capita disposable income of rural residents	0.1255
	Differences in local fiscal revenue	Urban general public finance revenue/ Rural general public finance revenue	0.0185
	Per capita production variance	Per capita GDP of secondary and tertiary industries / Per capita GDP of primary industry	0.0658
Social co-construction	Difference in medical level	Number of beds in medical and health institutions per 1,000 urban population / Number of beds in medical and health institutions per 1,000 rural population	0.0117
	Resident employment coordination degree	Urban employed population at the end of the year / Rural employment population at the end of the year	0.0316
	Coordination degree of urban and rural information infrastructure construction	Urban broadband access users / Rural broadband access users	0.0118
Cultural integration	Educational level difference	High amount of financial subsidy / Rural primary and junior high fiscal subsidy amount	0.0642
	Book resource difference	The number of public library books per capita in urban areas / Number of public library books per capita in rural areas	0.0230
	Cultural consumption difference	Urban residents’ expenditure on education, culture and entertainment/ Rural residents’ spending on education, culture and entertainment	0.0118
Ecological co-governance	Park green area difference	Per capita urban green space / Rural per capita green space	0.0123
	Water consumption variance	Urban per capita daily water consumption / Per capita daily water consumption in rural areas	0.1050
	Coordination degree of energy conservation and emission reduction	Urban per capita living carbon emission / Rural per capita living carbon emission	0.2092

The symbiosis model was used to quantitatively evaluate the symbiotic relationship between urban and rural areas. Among them, the symbiotic attribute of urban and rural areas is reflected by the qualitative parameter *Z*. In Eq (2), *n* is the total number of indicators, *W*_*i*_ is the weight of indicator *i*, and *Q*_*i*_ is the corresponding sample value.


$$Z=\sum_{i=1}^{n}W_{i}Q_{i}$$ (2)

The degree of symbiosis is used to describe the degree of correlation between quality parameters, and the symbiosis expression in this paper is shown in Eq (3):

$$\left\{\begin{array}{c} \alpha_{{\text{u}}_{i}c_{i}}=(\frac{dZ_{ui}}{Z_{ui}})/(\frac{dZ_{ci}^{}}{Z_{ci}})\\ \alpha_{c_{i}u_{i}}=(\frac{dZ_{ci}}{Z_{ci}})/(\frac{dZ_{ui}}{Z_{ui}}) \end{array}\right.$$ (3)

In Formula (3), *α*_*uici*_ and *α*_*ciui*_ respectively represent the degree of rural-urban symbiosis between city and township in place *i* and the degree of rural-urban symbiosis between town and city in place *i*. *Z*_*ui*_ and *Z*_*ci*_ respectively represent the qualitative parameters of cities and villages in place *i*. In addition, it can be seen from the scatter plot that there is a significant linear relationship between urban and rural quality parameters, so the linear relationship expression of urban and rural quality parameters is proposed as shown in Eq (4):

$$\left\{\begin{array}{c} Z_{\text{u}i}=\beta Z_{ci}+\gamma \\ Z_{ci}=\mu Z_{ui}+\delta \end{array}\right.$$ (4)

Collating (2)(3)(4) to obtain the expression of symbiosis in this paper is as follows:

αuici=(dZuiZui)/(dZciZci)=(dZuidZci)×(ZciZui)=βZciβZci+γαciui=(dZciZci)/(dZuiZui)=(dZcidZui)×(ZuiZci)=μZuiμZui+δ (5)

The measurement of the symbiosis coefficient *θ* is based on the results of the symbiosis degree, which can more intuitively reflect the degree of interaction and influence between urban and rural areas, and the specific expression is as follows.


$$\left\{\begin{array}{c} \theta_{u_{i}c_{i}}=\frac{\left|\alpha_{u_{i}c_{i}}\right|}{\left|\alpha_{u_{i}c_{i}}\right|+\left|\alpha_{c_{i}u_{i}}\right|}\\ \theta_{c_{i}u_{i}}\;=\;\frac{\left|\alpha_{c_{i}u_{i}}\right|}{\left|\alpha_{u_{i}c_{i}}\right|+\left|\alpha_{c_{i}u_{i}}\right|}\\ \theta_{u_{i}c_{i}}+\theta_{c_{i}u_{i}}=1 \end{array}\right.$$ (6)

When *θ*_*uici*_ = *θ*_*ciui*_ = 0.5, the role between urban and rural areas is balanced, the two develop in synergy, common prosperity, and the level of urban-rural symbiosis is the highest; When *θ*_*uici*_×*θ*_*ciui*_ = 0, only one party in urban or rural areas has developed, the other party has stagnated, and the level of urban-rural symbiosis is the lowest; When 0<*θ*_*ciui*_<*θ*_*uici*_<1, it showed the characteristics of biased urban development, urban benefits more than rural areas, and the level of urban-rural symbiosis is lower. When 0<*θ*_*uici*_ <*θ*_*ciui*_<1, it is characterized by a bias towards rural development, rural areas benefit more than cities, and the level of urban-rural symbiosis is low. To facilitate comparison, this paper moderately treats the symbiosis level to form a positive indicator in regression, and the specific calculation formula is as follows:

$$GSD=-|\theta_{\text{uici}}-0.5|$$ (7)

In Eq (7), *GSD* is the obtained urban-rural symbiosis level, and *θ*_*uici*_ is the city-rural symbiosis coefficient of *i*.

#### 4.2.3. Control variables

To reduce the endogenous problems caused by missing variables, this paper draws on existing research and takes economic development (*LNGDP*), industrial structure (*INDU*), opening up (*OPEN*), science and technology development (*TECH*), physical capital investment (*PHCI*), and population size (*LNPOPU*) as the control variables. Among them, the level of economic development is measured by per capita GDP [39]; The industrial structure is measured by the proportion of the added value of the secondary and tertiary industries to GDP [19]; Opening up to the outside world is measured by the proportion of total imports and exports to GDP [51]; The level of scientific and technological development is taken from the proportion of scientific and technological expenditure in fiscal expenditure [52]; Physical capital investment is expressed as the ratio of fixed asset investment to GDP of the whole society [39]; Population size is measured by the total population at the end of the year [52].

### 4.3. Data sources and descriptive statistical analysis

This paper uses panel data at the provincial level to study the relationship between the flow of elements between urban and rural areas and the symbiosis between urban and rural areas. Considering that the urbanization rate of the four municipalities directly under the central government of Beijing, Tianjin, Shanghai and Chongqing is close to 90%, and the boundary between urban and rural areas is not very clear [44], the sample selection is screened, and finally the panel data of 27 provinces and autonomous regions in China from 2010 to 2020 are selected as the research sample, and the data involved are derived from the China Statistical Yearbook, China Financial Yearbook, China Agricultural Statistical Yearbook, and the statistical yearbooks of various provinces and autonomous regions, etc., and the missing data of individual years in some provinces are filled in by linear interpolation. To eliminate the effect of heteroscedasticity, the natural logarithm of non-ratio variables (economic development level, population size) is taken, and the descriptive statistics of each variable are shown in Table 3 below.

**Table 3:** Descriptive statistics for each variable.

Variable name		Symbol	Sample size	Minimum value	Maximum value	Average value	Standard deviation	Median
Explained variable	Urban-rural symbiosis	CXGS	297	-0.413	-0.001	-0.119	0.090	-0.108
Explanatory variable	Total factor flow	QYS	297	0.121	0.938	0.503	0.151	0.504
	Factor flow of labor force	LABOUR	297	0.054	0.683	0.358	0.119	0.367
	Capital factor flow	CAPITAL	297	0.043	0.490	0.319	0.090	0.331
	Technical factor flow	SKILL	297	1.424	13.427	4.478	2.068	4.028
	Land factor flow	LAND	297	0.000	0.151	0.037	0.041	0.024
Control variable	Level of economic development	LNGDP	297	9.464	11.715	10.640	0.409	10.625
	Industrial structure	INDU	297	0.500	3.166	1.065	0.392	0.986
	Open to the outside world	OPEN	297	0.007	1.271	0.205	0.221	0.123
	Scientific and technological development	TECH	297	0.002	0.068	0.016	0.011	0.012
	Physical capital investment	PHCI	297	0.176	1.597	0.834	0.278	0.841
	Population size	LNPOPU	297	5.704	9.442	8.191	0.879	8.375
	Government intervention	GOVERN	297	0.106	1.379	0.290	0.221	0.229

## 5. Empirical analysis

### 5.1. Impacts of total factor flow on urban and rural symbiosis

To prevent the phenomenon of “pseudo-regression”, this paper uses the unit root test method ADF-Fisher to test the stationarity of each variable in the model, and the variables involved in this paper do not accept the null hypothesis at the 1% confidence level, so it can be assumed that each variable is a first-order monolith, that is, the measurement data of the variables involved in this paper are stable and can be added to the panel model for regression.

To avoid the bias of the estimation results due to the multicollinearity problem between the variables, this paper performs the variance inflation factor (VIF) test for each variable in the benchmark model. The maximum value of the VIF of each variable is 2.52, which is much lower than the critical value of 10, indicating that there is no multicollinearity problem between the variables.

#### 5.1.1. Baseline result analysis

Table 4 reports the core test results of the impact of total factor mobility level on the urban-rural symbiotic relationship. Woodridge [53] believes that it is best to use a fixed-effect model for macroscopic data, and the two-way fixed-effect model solves the problem of missing variables and endogenous factors that do not change with individuals and time by controlling individuals and time, making the model estimation results more robust [54]. Models (1), (2), (3) and (4) are two-way fixed-effect model test results. Models (1) and (2) test the relationship between total factor flow and urban-rural symbiosis from a linear perspective. Model (2) adds control variables based on model (1), and the total factor flow still positively affects the urban-rural symbiosis level, and the impact coefficient shows an increasing trend, indicating that from the overall linear point of view, the total factor flow has a significant positive effect on the urban-rural symbiosis level, and the larger the relative scale of factors in the countryside, the higher the urban-rural symbiosis level. Model (3) and Model (4) are tests of the relationship between total factor flow and urban-rural symbiosis from a nonlinear perspective. The primary term and quadratic term of the total factor flow in model (3) were significantly negative, the quadratic term was significantly positive, and the goodness-of-fit coefficients were improved after adding control variables in model (4), and the coefficients of primary term and quadratic term of total factor flow were -0.157 and 0.159, respectively, and both were significant at the 5% level, indicating that the U-shaped relationship between the total factor flow and urban-rural symbiosis. This conclusion is still tenable under the individual fixed-effect model of the model (5) and the random-effects model of the model (6).

**Table 4:** Regression results of total factor flow on urban-rural symbiosis level.

Variable	Model (1)	Model (2)	Model (3)	Model (4)	Model (5)	Model (6)
QYS	0.035^*^	0.039^**^	-0.169^**^	-0.157^**^	-0.218^***^	-0.219^***^
	(1.937)	(2.062)	(-2.215)	(-1.983)	(-3.207)	(-3.277)
QYS^2^			0.166^***^	0.159^**^	0.202^***^	0.202^***^
			(2.755)	(2.543)	(3.526)	(3.597)
LNGDP		-0.022		-0.017	0.074^***^	0.071^***^
		(-1.157)		(-0.891)	(11.365)	(12.618)
INDU		-0.003		-0.004	0.015^***^	0.017^***^
		(-0.407)		(-0.507)	(2.696)	(3.125)
OPEN		0.013		0.010	-0.018	-0.011
		(0.576)		(0.439)	(-0.874)	(-0.602)
TECH		-0.736^***^		-0.708^***^	-0.805^***^	-0.769^***^
		(-3.220)		(-3.125)	(-4.114)	(-3.978)
PHCI		0.005		0.007	0.012^**^	0.011^**^
		(0.851)		(1.212)	(2.415)	(2.277)
LNPOPU		0.044		0.051	0.002	0.041^***^
		(1.018)		(1.195)	(0.041)	(2.662)
CONS	-0.183^***^	-0.313	-0.126^***^	-0.37	-0.878^***^	-1.167^***^
	(-20.05)	(-0.91)	(-5.61)	(-1.09)	(-2.73)	(-9.57)
Individual	YES	YES	YES	YES	YES	NO
Time	YES	YES	YES	YES	NO	NO
*N*	297	297	297	297	297	297
R-sq	0.717	0.736	0.726	0.743	0.704	0.703

**Note:** *, ** and ***respectively indicate that they pass the test at the significance level of 10%, 5% and 1%; () is the value of T. The same below.

Furthermore, the paper resorts to the utest to analyze the U-shaped relationship between the total factor flow and urban-rural symbiosis and the test results are shown in Table 5. The extreme values were all within the sample data range of the total factor flow, and all within the confidence interval of 95% level under the Fieller standard error, and model (4), model (5) and model (6) all passed the U-shaped relationship test, which proved that the U-shaped nonlinear effect of "first inhibiting and then promoting" of the total actor flow on urban-rural symbiosis, reflecting that in the early stage of the all-factor (rural) flow, urban development was the main contradiction, and factors should flow to cities with higher utilization efficiency. Otherwise, there will be a situation in which the flow of factors (to the countryside) inhibits the symbiotic development of urban and rural areas; When the flow of factors (to the countryside) breaks through the extreme inflection point, rural development becomes the main contradiction, and the factors should be concentrated in the countryside, which is conducive to promoting the improvement of the level of urban-rural symbiosis, assuming that H1 is established.

**Table 5:** Test of U-shaped relationship between urban and rural symbiosis level by total factor flow.

	Model (4)	Model (5)	Model (6)
U-shaped test t value	1.84	3.10	3.17
U-shaped test P value	0.034	0.001	0.001
Extreme point	0.494	0.540	0.542
Confidence interval	[0.014;0.609]	[0.424;0.623]	[0.430;0.623]
Slope on both sides of curve	-0.119,0.142	-0.169 ,0.161	-0.171,0.160
The slope P on both sides of the curve	0.034,0.001	0.001,0.000	0.001,0.000
Relationship	U-shape	U-shape	U-shape

China’s urban and rural development is largely influenced by the country’s macro policy orientation [55]. The sample inspection period began in China’s "Twelfth Five-Year" planning period, In 2011, China’s urbanization rate exceeded 50%, heralding the city-based social development model. Under the guidance of “urbanization with Chinese characteristics”, the government seeks urban sprawl with focus on industrial development and land management, transforming the rural land into the urban construction land, resulting in the centralized allocation of the technology, capital, among other elements, and sending the labor force to urban areas At the moment, the level of all factors in the countryside is low, and the overall development of society, including the countryside, is mainly driven by the city, and the urban-rural relationship is characterized by a high level of urban-rural symbiosis, At this stage, if the level of all factors in the countryside is continuously improved, and the various resource elements invested in urban development are decreased,, the advantages of urban resource utilization efficiency will be weakened, and the ability of cities to drive the overall development of society including rural areas will be reduced, resulting in a decrease in the level of urban-rural symbiosis; Until the lowest point (inflection point) of urban-rural symbiosis level, it is manifested as the slow development of the city, such as rapid urbanization causing problems such as traffic congestion, industrial hollowing, and environmental pollution in the city [56], and the city’s own ability is difficult to effectively drive rural development, and the countryside is completely stagnant; When the total factor breaks through the extreme value of the inflection point at the rural level, the rural economy can be restored with the continuous support of resource factors, and the ability of independent development is cultivated, This is the stage when rural areas enjoy self-driven development and fuel urban development, and the level of urban-rural symbiosis can be continuously improved. Just as the Central Document No.1 of 2018 clearly proposed to implement the "rural revitalization strategy", society began to pay attention to the problem of cities feeding the development of the countryside, comprehensively revitalizing the countryside, and solving the long-term problem of insufficient rural development has become the pressing task. Based on the estimation results of the double fixed-effect model of model (4), the flow level of total factors (to the countryside) at the inflection point of the "U-shaped" curve is about 0.494, and the median and average in the flow of total factors (to the countryside) in 27 provinces (autonomous regions) in China during the investigation period are 0.503 and 0.504, respectively, which can be found are located on the right side of the inflection point of the "U-shaped" curve, indicating that the flow level of total factors (to the countryside) in more than half of the regions in China during the investigation period crossed the development inflection point and entered the stage of promoting the symbiotic development of urban and rural areas through the flow level of factors (to the countryside). The time has come for cities to feed the development of the countryside.

#### 5.1.2. Robustness analysis

In this paper, the robustness of the benchmark regression results is tested from four aspects. First, increase the control variable. In China, the flow direction and intensity of total factors between urban and rural areas are mostly the result of macro policy regulation [55]. With the implementation of the new idea of urban-rural integration, more public financial resources are constantly given to rural areas, and the impact of government intervention on urban-rural symbiosis cannot be ignored. Based on existing research [19], this paper uses the ratio of government fiscal expenditure to GDP to measure the degree of government intervention (*GOVERN*)and further verifies the impact of total factor flow on urban-rural symbiosis by adding this control variable. Data from China Statistical Yearbook (2011–2021). From the regression results of Model 1 in Table 6, the primary and secondary terms of total factor flow after adding the control variables are significantly negative and positive at the 5% level, respectively, which is consistent with the benchmark nonlinear conclusion. Second, all explanatory variables lag by one period. The influence of total factor flow on urban-rural symbiosis may have a time lag, so this paper performs double-fixed-effect model regression for the lagging first-phase regression of all explanatory variables, and the results are shown in the model (2). The primary term of total factor flow is significantly negative at the 1% level, and the quadratic term is significantly positive at the 1% level, which is consistent with the benchmark regression results. Third, eliminate extreme values. To avoid the influence of outliers on the study results, Winsorize tail shrinkage was performed on 1% and 99% quantiles for all continuous variables, and a two-way fixed-effect model was repeated for benchmark regression. The estimation results are shown in model (3). The coefficient symbols and significance of the primary and quadratic terms of the total factor flow are the same as the benchmark results, which fully indicates that the results of the benchmark regression are robust and reliable. Fourth, endogenous discussions. Although the relevant variables, time and year fixed effects are controlled, the Hausman test rejects the null hypothesis at the 1% level and proves that the model still has endogenous problems, according to which, to ensure the accuracy of the model measurement results, this paper refers to the practice of Yang Jin [57], uses the first-order lag term of the total factor flow as a tool variable, and estimates by the two-stage least squares method (2SLS) of the individual fixed and bidirectional fixed panels respectively, and the results are shown in the model (4) and model (5). On the one hand, the flow of total factors is related to its first-order lag term, that is, it meets the correlation requirements of instrumental variables. On the other hand, compared with the current variable, the first-order lagged term of the total factor flow has occurred, which is a predetermined variable, that meets the exogenous requirements of the instrument variable and the random perturbation term, and the probability values of the F statistic are significant at the level of 5%, which proves that there is no weak correlation problem. In summary, the sign and significance of the parameter estimation results did not change under different test methods, so the research conclusions of the previous benchmark model are reliable.

**Table 6:** Robustness and endogeneity tests.

Variable	Add control variable	Explanatory variables lag by one phase	Bidirectional tail shrinkage 0.01	Panel 2SLS	Panel 2SLS
	Model (1)	Model (2)	Model (3)	Model (4)	Model (5)
QYS	-0.157^**^	-0.162^***^	-0.146^*^	-0.492^***^	-0.531^***^
	(-1.974)	(-2.676)	(-1.88)	(-3.31)	(-3.00)
QYS^2^	0.159^**^	0.137^***^	0.144^**^	0.439^***^	0.464^***^
	(2.524)	(2.730)	(2.34)	(3.45)	(3.14)
LNGDP	-0.017	0.018^*^	-0.018	0.07^***^	0.001
	(-0.835)	(1.964)	(-0.93)	(9.64)	(0.04)
INDU	-0.004	0.003	-0.005	0.01^**^	-0.006
	(-0.505)	(0.523)	(-0.67)	(1.98)	(-0.79)
OPEN	0.009	-0.007	0.017	-0.004	0.006
	(0.437)	(-0.475)	(0.83)	(-0.21)	(0.30)
TECH	-0.708^***^	-0.772^***^	-0.786^***^	-0.589^***^	-0.499^***^
	(-3.118)	(-3.827)	(-3.41)	(-3.61)	(-2.58)
PHCI	0.007	0.021^***^	0.007	0.005	0.009^*^
	(1.208)	(3.550)	(1.27)	(1.00)	(1.65)
LNPOPU	0.051	-0.002	0.075^*^	0.082^*^	0.106^**^
	(1.192)	(-0.679)	(1.76)	(1.79)	(2.44)
GOVERN	0.001				
	(0.023)				
CONS	-0.372	-0.583^*^	-0.554	-1.464^***^	-0.905
	(-1.05)	(-1.84)	(-1.63)	(-4.01)	(-2.55)
Individual	YES	YES	YES	YES	YES
Time	YES	YES	YES	NO	YES
N	297	296	297	270	270
R-sq	0.743	0.754	0.749	0.977	0.980

### 5.2. The impact of single factor flow on urban and rural symbiosis

From the above empirical regression results, it can be seen that the flow level of total factors has a U-shaped nonlinear influence on the urban-rural symbiotic relationship. Considering that total factor flow is a comprehensive indicator, the degree of influence of labor, capital, technology, land and other factors on the symbiotic relationship between urban and rural areas may be different. Therefore, starting from the four dimensions of total factor flow, based on the double fixed effect model, this paper will examine the effects of labor factor flow, capital factor flow, technology factor flow, and land factor flow on urban-rural symbiotic relationship from linear and nonlinear perspectives, and the results are shown in Table 7.

**Table 7:** Influence of single factor flow on urban-rural symbiosis.

Variable	Model (1)	Model (2)	Model (3)	Model (4)	Model (5)	Model (6)	Model (7)	Model (8)
LABOUR	-0.159^***^	0.491^***^						
	(-4.003)	(4.855)						
LABOUR^2^		-0.687^***^						
		(-6.893)						
CAPTIAL			0.077^***^	-0.221^**^				
			(3.067)	(-2.363)				
CAPTIAL^2^				0.565^***^				
				(3.299)				
SKILL					0.001	-0.001		
					(0.476)	(-0.327)		
SKILL^2^						0.000		
						(0.510)		
LAND							-0.980^***^	0.002
							(-3.924)	(0.005)
LAND^2^								-6.159^**^
								(-2.582)
LNGDP	-0.033^*^	-0.080^***^	-0.016	-0.026	-0.013	-0.009	-0.001	-0.012
	(-1.760)	(-4.322)	(-0.888)	(-1.409)	(-0.675)	(-0.418)	(-0.049)	(-0.664)
INDU	-0.005	-0.010	0.003	-0.001	-0.001	0.000	0.002	-0.002
	(-0.599)	(-1.476)	(0.405)	(-0.188)	(-0.096)	(0.007)	(0.295)	(-0.216)
OPEN	-0.020	-0.058^***^	0.003	-0.000	0.006	0.004	-0.013	-0.027
	(-0.918)	(-2.756)	(0.139)	(-0.015)	(0.271)	(0.201)	(-0.607)	(-1.217)
TECH	-0.829^***^	-0.416^*^	-0.778^***^	-0.842^***^	-0.782^***^	-0.788^***^	-0.901^***^	-0.892^***^
	(-3.729)	(-1.952)	(-3.456)	(-3.800)	(-3.405)	(-3.422)	(-4.018)	(-4.019)
PHCI	0.006	0.007	0.005	0.003	0.007	0.007	0.004	0.005
	(1.023)	(1.277)	(0.847)	(0.500)	(1.070)	(1.087)	(0.714)	(0.762)
LNPOPU	-0.047	0.024	0.041	0.065	0.027	0.028	0.029	0.022
	(-1.042)	(0.575)	(0.972)	(1.559)	(0.634)	(0.647)	(0.701)	(0.531)
CONS	0.636	0.400	-0.353	-0.416	-0.256	-0.298	0.348	-0.185
	(1.60)	(1.09)	(-1.04)	(-1.25)	(-0.74)	(-0.84)	(-1.04)	(-0.55)
Individual	YES	YES	YES	YES	YES	YES	YES	YES
Time	YES	YES	YES	YES	YES	YES	YES	YES
*N*	297	297	297	297	297	297	297	297
R-sq	0.748	0.788	0.742	0.752	0.732	0.733	0.747	0.754

In model (1), the primary term of labor factor flow is significantly negative, indicating that the flow of labor factors to the countryside from a linear perspective will inhibit urban-rural symbiosis. In model (2), the primary term coefficient of labor factor flow is positive, the quadratic term coefficient of it is negative, both of them exceed the significance level of 1%, and the goodness-of-fit of the nonlinear model is higher than that of the linear model, indicating that overall, there is a significant inverted U-shaped relationship between labor factor flow and urban-rural symbiosis. The inflection point of the curve is 0.357, and the average and median of the national labor factor flow are 0.358 and 0.367, respectively, slightly higher than the inflection point level. The right side of the inverted U-shaped curve revealed that the level of labor factor (to the countryside) flow in half of the regions has broken through the inflection point and entered the stage of inhibiting the symbiotic development of urban and rural areas, that is, the transfer of labor factors to the countryside is not the policy preference in some areas, if the urban development in these areas is not sufficient, the advantageous areas of resource utilization efficiency are still in the city rather than the countryside. Perhaps county urbanization, agricultural transfer population near urbanization, with rural industrialization, scale, and intensive development is a better way to improve the level of urban-rural symbiosis.

Model (3) reveals that the flow of capital factors to rural areas from a linear perspective has a significant positive effect on the level of urban-rural symbiosis. Model (4) shows that the goodness-of-fit of the nonlinear model is improved, and the primary term of capital factor flow is significantly negative at the 5% level, and the quadratic term is significantly positive at the 1% level, indicating that there is a U-shaped nonlinear relationship between the two, and the capital factor flow level at the inflection point of the curve is 0.196, while the average number of capital factor flow in the country is 0.319. The median is 0.331, which is much higher than the inflection point level, and is located on the right side of the U-shaped curve, indicating that most of the country has crossed the inflection point of capital factor flow and entered the stage where capital flow to rural areas plays a positive role in promoting urban-rural symbiosis, which can further encourage "capital to go to the countryside". Model (5) and (6) shows that the primary and quadratic coefficients of the flow of technical elements are not significant, that is, the flow of technical factors has no obvious effect on the level of urban-rural symbiosis.

From a linear point of view, the coefficient of land element flow in the model (7) is -0.980 and is significant at the level of 1%, which indicates that the flow of land factors has a significant negative impact on the level of urban-rural symbiosis. From the perspective of nonlinearity, the primary term of land element flow in the model (8) was not significant, and the quadratic term was significantly negative at the 5% level, indicating that there was a significant inverted U-shaped relationship between the two. The inflection point is 0.000, the median of the national land element flow is 0.024, and the average is 0.037, which is much higher than the inflection point level and is located on the right side of the inverted U-shaped curve, indicating that the flow of land elements to the city has broken through the inflection point, and most areas of the country have entered the stage of inhibiting the flow of land elements to urban and rural coexistence, and consideration should be given to limiting the scale of urbanization land, protecting rural land areas and cultivated areas, and promoting the sustainable and coordinated development of urban and rural areas.

### 5.3. Regional heterogeneity analysis

#### 5.3.1. Regional heterogeneity analysis of total factor flow affecting urban-rural symbiosis level

Due to the differences in geographical location, economic foundation and resource endowment, the total factor flow and urban-rural symbiosis level are not balanced, and there are obvious regional differences. In this paper, a double-fixed-effect model is used to explore the regional differences in the influence of total factor flow on urban-rural symbiotic relationships, and the regression results are shown in Table 8. Among them, models (1) and model (2) are the regression results of the more developed regions including Hebei, Jiangsu, Zhejiang, Fujian, Shandong, Guangdong, and Hainan Eastern Provinces, and models 3 and 4 are the estimation results of the less developed regions in 20 central, western and northeastern provinces (autonomous regions) except the east.

**Table 8:** Regional heterogeneity of total factor mobility on urban-rural symbiosis level.

Variable	Relatively developed area		Underdeveloped area	
	Model (1)	Model (2)	Model (3)	Model (4)
QYS	-0.025	-0.007	0.02	-0.242^**^
	(-0.77)	(-0.07)	(0.91)	(-2.47)
QYS^2^		-0.013		0.219^***^
		(-0.19)		(2.74)
LNGDP	-0.031	-0.03	-0.045^*^	-0.035
	(-1.10)	(-1.04)	(-1.90)	(-1.49)
INDU	0.041^***^	0.041^***^	-0.016	-0.016
	(4.72)	(4.67)	(-1.57)	(-1.61)
OPEN	-0.05^*^	-0.048^*^	-0.118^**^	-0.104^**^
	(-1.91)	(-1.77)	(-2.46)	(-2.20)
TECH	-0.203	-0.196	-1.148^***^	-1.045^***^
	(-0.88)	(-0.83)	(-3.78)	(-3.47)
PHCI	0.018	0.018	0.006	0.008
	(1.04)	(1.00)	(0.93)	(1.17)
LNPOPU	0.083	0.083	0.053	0.067
	(1.06)	(1.04)	(1.03)	(1.32)
CONS	-0.505	-0.519	-0.135	-0.28
	(-0.71)	(-0.72)	(-0.33)	(-0.69)
Individual	YES	YES	YES	YES
Time	YES	YES	YES	YES
*N*	77	77	220	220
R-sq	0.867	0.867	0.770	0.779

From the regression results of more developed regions, the regression coefficients of the primary and secondary terms of the total factor flow level in model (1) and model (2) were not significant, indicating that the total factor flow level in the eastern developed region had no significant impact on urban-rural symbiosis. The possible reason is that developed regions take advantage of their location advantages on the eastern coast to take the lead in development, becoming the main distribution of China’s megacities and megacities, maintaining a leading position in the country in terms of industrial upgrading, ecological governance, local financial resources, etc., and the entire eastern coastal area has formed a regional continuous urban economic belt, earlier realized rural industrialization, peasant urbanization and non-agricultural employment [58], its urbanization road is endogenous [59], the urbanization rate of developed eastern regions such as Guangdong, Jiangsu and Zhejiang has crossed the 70% mark [60], the degree of urban-rural integration is high, the boundary between urban and rural is gradually blurred, the production factors gradually tend to be balanced between urban and rural areas, the difference in the scale of factors after the flow of all factors is small, and the level of urban-rural symbiosis is high, so the relationship between the two is not obvious.

From the regression results of the underdeveloped areas, the coefficient of the primary term of the total factor flow level in model (3) was not significant, the primary term of the total factor flow level in model (4) was significantly negative at the 5% level, and the quadratic term was significantly positive at the 1% level, and compared with the linear model, the goodness of fit of the nonlinear model was improved, indicating that the linear influence of the total factor flow level on urban-rural symbiosis in the underdeveloped area was not obvious, and it mainly showed a significant U-shaped nonlinear influence. The inflection point value was 0.553, and the average and median of total factor flow in underdeveloped areas were 0.503 and 0.504, which was lower than the inflection point value, indicating that the total factor flow level in most underdeveloped areas had not yet broken through the inflection point, and was in the stage where the level of factor to rural mobility inhibited the symbiotic development of urban and rural areas. Most of the underdeveloped areas in the central and western regions are sparsely populated, due to the lack of location advantages and missed opportunities for rural industrialization, there is no longer the possibility of re-rural industrialization [55], 80% of the country’s farmers and more than 70% of agricultural productivity are concentrated, its urbanization is dotted [47], urban development is far from enough, it is difficult to drive the comprehensive development of the entire region, including the countryside, so the main contradiction in these areas to solve the problem of urban-rural symbiotic development is in the city, and the factors should flow more to the city, gather and gather, with the city with the countryside. Promote common prosperity in urban and rural areas.

#### 5.3.2. Regional heterogeneity analysis of single factor flow affecting urban-rural symbiosis level

The differential influence of the flow level of various factors on the urban-rural symbiosis in the developed areas is deconstructed, and the regression results are shown in Table 9. Models (1) and (2) show that, from a linear perspective, the flow of labor factors to rural areas has an inhibitory effect on urban-rural symbiosis at the 1% level. From the perspective of nonlinear, the primary term and the quadratic term of labor flow are significantly positive and negative respectively, and its relationship with urban and rural symbiosis presents an inverted U-shaped relationship. The inflection point value of the factor level is 0.132, the average level of labor mobility to rural areas in developed areas is 0.241, and the median is 0.244, both higher than the inflection point value and located on the right side of the inverted U-shaped curve. In other words, the flow of the labor force to rural areas in most developed areas inhibits the symbiotic development of urban and rural areas, so the labor force should be transferred to more efficient urban areas or continue to promote the process of urbanization, local urbanization of agricultural population transfer, continue to accelerate the urbanization rate in more developed areas, and promote the urban-rural development in the region. In models (3) and (4), the coefficients of the first and second terms of capital factor flow are not significant, that is, the capital factor flow in the more developed areas does not influence the urban-rural symbiosis.

**Table 9:** Influence of factor flow levels on urban-rural symbiosis in developed areas.

Variable	Model (1)	Model (2)	Model (3)	Model (4)	Model (5)	Model (6)	Model (7)	Model (8)
LABOUR	-0.606^***^	0.251^*^						
	(-7.266)	(1.407)						
LABOUR^2^		-0.951^***^						
		(-5.202)						
CAPITAL			0.025	-0.125				
			(0.890)	(-1.307)				
CAPITAL^2^				0.328				
				(1.630)				
SKILL					-0.002	-0.020^***^		
					(-1.030)	(-2.690)		
SKILL^2^						0.001^**^		
						(2.524)		
LAND							-0.712^***^	2.490^***^
							(-3.191)	(5.118)
LAND^2^								-14.142^***^
								(-6.981)
LNGDP	-0.062^***^	-0.105^***^	-0.035	-0.042	-0.038	-0.011	-0.020	-0.061^***^
	(-3.155)	(-5.818)	(-1.291)	(-1.548)	(-1.418)	(-0.379)	(-0.790)	(-3.159)
INDU	0.012	-0.019^**^	0.040^***^	0.035^***^	0.040^***^	0.036^***^	0.046^***^	0.026^***^
	(1.635)	(-2.241)	(4.612)	(3.778)	(4.561)	(4.271)	(5.607)	(3.902)
OPEN	-0.093^***^	-0.085^***^	-0.040	-0.029	-0.041	-0.063^**^	-0.054^**^	-0.090^***^
	(-4.934)	(-5.445)	(-1.572)	(-1.112)	(-1.654)	(-2.498)	(-2.356)	(-5.121)
TECH	-0.329^*^	-0.071	-0.201	-0.204	-0.165	-0.409^*^	-0.342	-0.254
	(-1.999)	(-0.492)	(-0.874)	(-0.903)	(-0.711)	(-1.695)	(-1.580)	(-1.612)
PHCI	0.011	-0.006	0.020	0.017	0.014	0.005	0.011	0.001
	(0.921)	(-0.596)	(1.187)	(1.042)	(0.777)	(0.281)	(0.722)	(0.127)
INPOPU	-0.042	0.208^***^	0.075	0.080	0.079	0.125	0.011	-0.148^**^
	(-0.725)	(3.074)	(0.961)	(1.040)	(1.011)	(1.636)	(0.146)	(-2.511)
CONS	1.129^**^	-0.69	-0.418	-0.378	-0.396	-1.006	0.049	1.746^***^
	(2.077)	(-1.22)	(-0.60)	(-0.54)	(-0.56)	(-1.41)	(0.07)	(3.23)
Individual	YES	YES	YES	YES	YES	YES	YES	YES
Time	YES	YES	YES	YES	YES	YES	YES	YES
*N*	77	77	77	77	77	77	77	77
R-sq	0.933	0.956	0.867	0.874	0.868	0.882	0.887	0.942

In model (5), the primary coefficient of technological factor flow is not significant, indicating that the linear influence of technological factor flow on the urban-rural symbiosis is not obvious. Model (6) shows that technological factor flow has a U-shaped influence on the urban-rural symbiosis level, the inflection point of factor level is 10.000, and the average and median of technological factor flowing to rural areas from more developed areas is 4.867 and 4.667. It is lower than the inflection point and located on the left side of the U-shaped curve, indicating that the flow of technological factors to rural areas in most developed areas inhibits the symbiotic development of urban and rural areas, so it is necessary to increase the input of factors in urban areas to promote the common development of rural areas.

There is an inverted U-shaped relationship between land factor flow and urban-rural symbiosis. The inflection point of land factor flow is 0.088, and the mean value and median value of land factor flow to the city in more developed areas are 0.085 and 0.094. Near the inflection point, the influence of land factor flowing to the city on urban-rural symbiosis is at the highest level, and this equilibrium state can be maintained.

The differential influence of the flow level of various factors on the urban-rural symbiosis in underdeveloped areas is deconstructed, and the regression results are shown in Table 10. The inflection point value of the factor level is 0.349. The average level of labor flow to rural areas in underdeveloped areas is 0.399, and the median is 0.387, which is higher than the inflection point value and located on the right side of the inverted U-shaped curve, indicating that the transfer of labor force to rural areas in more than half of underdeveloped areas inhibits the improvement of urban-rural symbiosis. These areas should also give priority to urban development and achieve common development through urban and rural development. The flow of capital factors has a U-shaped influence on the level of urban and rural symbiosis. The inflection point of the factor level is 0.255, and the mean and median of the flow of capital factors to rural areas in underdeveloped areas are 0.331 and 0.341, which are higher than the inflection point and located on the right side of the U-shaped curve, indicating that in underdeveloped areas, capital factors continue to transfer to rural areas, contributing to the improvement of the level of urban-rural symbiosis. Achieve common prosperity and progress in both urban and rural areas. The other two factors, the flow of land and technology, have no obvious influence on the urban-rural symbiosis in underdeveloped areas.

**Table 10:** Influence of factor flow level on urban-rural symbiosis in underdeveloped areas.

Variable	Model (1)	Model (2)	Model (3)	Model (4)	Model (5)	Model (6)	Model (7)	Model (8)
LABOUR	-0.118^***^	0.350^***^						
	(-2.785)	(2.920)						
LABOUR^2^		-0.504^***^						
		(-4.144)						
CAPITAL			0.034	-0.446^***^				
			(0.994)	(-3.435)				
CAPITAL^2^				0.876^***^				
				(3.815)				
SKILL					0.001	-0.004		
					(0.872)			
SKILL^2^						0.000		
						(1.205)		
LAND							-0.515	-1.340^*^
							(-1.366)	(-2.059)
LAND^2^								10.143
								(1.552)
LNGDP	-0.057^**^	-0.090^***^	-0.042^*^	-0.051^**^	-0.043^*^	-0.032	-0.034	-0.030
	(-2.462)	(-3.835)	(-1.851)	(-2.323)	(-1.886)	(-1.307)	(-1.463)	(-1.269)
INDU	-0.018^*^	-0.022^**^	-0.012	-0.016	-0.015	-0.011	-0.014	-0.014
	(-1.821)	(-2.321)	(-1.172)	(-1.596)	(-1.536)	(-1.105)	(-1.386)	(-1.437)
OPEN	-0.137^***^	-0.136^***^	-0.122^**^	-0.171^***^	-0.118^**^	-0.114^**^	-0.133^***^	-0.117^*^
	(-2.977)	(-3.071)	(-2.589)	(-3.619)	(-2.460)	(-2.369)	(-2.844)	(-2.438)
TECH	-1.212^***^	-0.788^***^	-1.175^***^	-1.283^***^	-1.161^***^	-1.134^***^	-1.210^***^	-1.220^**^
	(-4.129)	(-2.630)	(-3.923)	(-4.418)	(-3.854)	(-3.757)	(-4.053)	(-4.104)
PHCI	0.007	0.009	0.007	0.004	0.007	0.008	0.007	0.008
	(1.141)	(1.410)	(1.080)	(0.613)	(1.082)	(1.142)	(0.980)	(1.172)
LNPOPU	-0.014	0.015	0.053	0.095	0.050	0.065	0.040	0.038
	(-0.255)	(0.281)	(1.030)	(1.873)	(0.987)	(1.235)	(0.802)	(0.746)
CONS	0.586	0.587	-0.165	-0.339	-0.127	-0.343	-0.123	-0.138
	(1.26)	(1.32)	(-0.40)	(-0.85)	(-0.32)	(-0.78)	(-0.31)	(-0.34)
Individual	YES	YES	YES	YES	YES	YES	YES	YES
Time	YES	YES	YES	YES	YES	YES	YES	YES
N	220	220	220	220	220	220	220	220
R-sq	0.781	0.799	0.772	0.789	0.772	0.774	0.774	0.777

## 6. Conclusion and suggestion

The full flow of urban and rural factors is the key to solving the integrated development of urban and rural areas in China. Based on the theory of ecological symbiosis, this paper interprets the new urban-rural relationship. It constructs a comprehensive evaluation index system for urban-rural symbiosis based on the concept of "political sharing, economic co-prosperity, social co-construction, cultural integration, and ecological co-governance". Based on China’s provincial panel data from 2010 to 2020, taking 27 provinces (autonomous regions) in China as the research objects, an urban-rural symbiosis model was constructed, and the level of urban-rural symbiosis in China was measured. The impact of all-factor flow and single-factor flow on urban-rural symbiosis level was empirically verified from the perspective of all factors of labor, capital, technology and land by using the panel double fixed effect model, and the regional differences of urban-rural symbiosis under all-factor flow and single-factor flow were discussed. The level of urban-rural integrated development was quantitatively evaluated with symbiosis coefficients. This paper expands the multi-dimensional analysis perspective of factor urban-rural flow, deconstructs the internal heterogeneity of factor flow on the development of the urban-rural symbiotic relationship, empirically tests the U-shaped effect relationship of factor flow on urban-rural symbiotic development, and gives the basic direction of classification policies to promote urban-rural integration and symbiotic development in different regions. The specific conclusions and recommendations are as follows:

First, the flow of all factors presents a significant U-shaped non-linear feature of urban-rural symbiosis. The level of all-factor (to the countryside) flow in more than half of China has crossed the inflection point of development and entered the stage of promoting the symbiotic development of urban and rural areas by the level of factor (to the countryside) flow. The time has come for cities to feed back the countryside.

Second, there are significant differences in the impact of different factor flows on urban-rural symbiosis. Specifically, the impact of the flow of labor factors to rural areas on urban-rural symbiosis is inverted U-shaped, and the level of labor factors (to rural areas) flow in half of the regions has broken through the inflection point and entered the stage of inhibiting the symbiotic development of urban and rural areas. The impact of capital factor flow and urban-rural symbiosis has a positive U-shaped relationship, and most of the country has crossed the inflection point of capital factor flow and entered the stage of positive promotion, which can further encourage "capital to flow to the countryside"; The impact of land factor flow on urban-rural symbiosis presents a significant inverted U-shaped relationship, and the flow of land elements to urban in most areas of the country has broken through the inflection point and entered the stage of inhibition, and consideration should be given to limiting the scale of urbanization land, protecting rural land areas and cultivated land areas, and promoting the sustainable and coordinated development of urban and rural areas. The impact of the flow of technological factors on the symbiotic development of urban and rural areas is not significant.

Third, there is significant regional heterogeneity in the impact of factor flow on the level of urban-rural symbiosis. The total factor flow in more developed areas does not affect the symbiosis between urban and rural areas; The impact of total factor flow in underdeveloped areas on urban-rural symbiosis is a significant U-shaped feature, and the total factor flow level in most underdeveloped areas has not yet broken through the inflection point and is in the stage of inhibiting development. In more developed areas, the level of labor flow and the symbiotic relationship between urban and rural areas are inverted U-shaped, and they are currently in the stage of inhibiting development, labor should be invested in more efficient urban areas or continue to promote the process of urbanization, driving the continuous development of urban and rural areas in the region. The flow of technical elements has a U-shaped impact on the level of urban-rural symbiosis, and the flow of technical elements to rural areas in most of the more developed areas inhibits the development of urban-rural symbiosis, and the investment of technical elements in urban areas can continue to increase. The flow of land factors and urban-rural symbiosis has an inverted U-shaped relationship, and the impact of land factor flow to urban on urban-rural symbiosis is at the highest level, which can maintain this equilibrium state. The flow of capital factors has no impact on the symbiotic relationship between urban and rural areas. In the less developed areas, the level of labor flow and the urban-rural symbiosis are in an inverted U-shaped relationship, which is currently in the stage of suppression, and urban development should be the top priority in these areas. The flow of capital factors has a U-shaped impact on the level of urban-rural symbiosis, and the current transfer of capital factors to rural areas is conducive to the improvement of urban-rural symbiosis and the realization of common prosperity and progress between urban and rural areas. The flow of land and technology does not have a significant impact on the symbiosis between urban and rural areas in less developed areas.

## Supporting information

S1 File (ZIP)
